# Characterisation of Syncytiotrophoblast Vesicles in Normal Pregnancy and Pre-Eclampsia: Expression of Flt-1 and Endoglin

**DOI:** 10.1371/journal.pone.0056754

**Published:** 2013-02-20

**Authors:** Dionne S. Tannetta, Rebecca A. Dragovic, Chris Gardiner, Christopher W. Redman, Ian L. Sargent

**Affiliations:** Nuffield Department of Obstetrics & Gynaecology, John Radcliffe Hospital, University of Oxford, Oxford, United Kingdom; Institute of Zoology, Chinese Academy of Sciences, China

## Abstract

**Background:**

The placental syncytiotrophoblast releases micro and nanovesicles (STBM), into the maternal circulation in normal pregnancy and in increased amounts in pre-eclampsia (PE), which have proinflammatory and antiangiogenic activity and are implicated in PE pathophysiology. Better characterisation of STBM is essential to understand their role in PE.

**Methods and Results:**

STBM prepared by placental lobe dual perfusion (pSTBM) and mechanical disruption (mSTBM) were analysed by four colour flow cytometry (4CFC), nanoparticle tracking analysis (NTA) and Western blotting to determine vesicle size, purity and Flt-1 and endoglin (Eng) expression. Biological activity of STBM associated Flt-1 and endoglin was assessed by the ability of VEGF, PlGF and TGFβ to bind to mSTBM and inhibit mSTBM induced endothelial monolayer disruption. STBM content was consistently high (∼87–95%) across the different preparations. However, surface antigen intensities differed, with significantly lower placental alkaline phosphatase (P<0.05) and Eng (P<0.05) expression on mSTBM, and Flt-1 (P<0.05) expression on pSTBM. For PE placenta derived preparations, pSTBM contained lower Eng positive STBM (P<0.05) and mSTBM Eng expression was increased (P<0.05). Western blotting revealed increased Flt-1/sFlt-1 (P<0.02) and decreased placental alkaline phosphatase (P = 0.0002) content of PE placenta pSTBM. Using NTA, perfused PE placentas released significantly larger MV (P<0.001). Finally, VEGF, PlGF and TGFβ bound to mSTBM at physiologically relevant concentrations and inhibited mSTBM induced endothelial disruption (P<0.05-P<0.001).

**Conclusions:**

This study has found differences in physical and antigenic characteristics of normal and PE placenta STBM preparations produced by placental perfusion or mechanical disruption. We have also demonstrated that large quantities of biologically active STBM associated endoglin and Flt-1/sFlt-1 could contribute to the increased circulating levels measured in PE patients and add to the perturbation of the maternal vascular endothelium, normally attributed to non-membrane bound sFlt-1 and sEndoglin.

## Introduction

Pre-eclampsia (PE) is a complex disorder of human pregnancy, which causes maternal and perinatal mortality or morbidity, and has long-term health implications for mother and surviving off-spring [1], [2]. Its first (pre-clinical) stage comprises deficient remodeling of the utero-placental circulation (8–18 weeks), dysfunctional perfusion and placental oxidative stress [3], [4]. The second (clinical) stage (after 20 weeks) results from systemic vascular inflammation. This has been shown to be an extension of a broader maternal systemic inflammatory response intrinsic to normal pregnancies, but more severe in pre-eclampsia, including endothelial dysfunction, and metabolic, clotting and complement disturbances. In searching for the cause of these changes in the mother in PE, our attention has focused on the role of syncytiotrophoblast derived vesicles (STBM). These are membrane bound vesicles shed from the syncytial epithelium (STB) of the placenta, that circulate during normal pregnancy and in significantly increased amounts in PE [5], [6].

Increasing evidence shows that STBM have functions relevant to PE. We and others have shown that they bind to, and are taken up by monocytes (both *in vivo* and *in vitro*), and stimulate the production of proinflammatory cytokines [7]–[10], activate neutrophils [11],[12], inhibit endothelial cell proliferation and tube formation or disrupt their growth as a monolayer [13]–[15], and inhibit the relaxation of pre-constricted blood vessels [16]. We have also shown that biologically active tissue factor on STBM triggers thrombin generation and that PE STBM have significantly higher TF levels than normal [17]. As well as having damaging effects, STBM can also down-regulate maternal immune responses. We and others have shown that STBM from normal placentas inhibit T [18], [19] and NK cell responses *in vitro* [20]. In order to understand the role of STBM in the maternal syndrome of PE, we need to be able to characterise them more fully.

Since our initial observations of circulating STBM in normal pregnancy and PE, the study of cellular vesicles in the biomedical field has grown exponentially, with the discovery of multiple types of cellular vesicles and their implication in a growing number of diseases [21]. In general vesicles can be categorised as being either microvesicles (MV: 100 nm –1 µm in diameter) which directly bud from the plasma membrane and are released in response to cell activation and death (apoptotic and necrotic) or nanovesicles (exosomes 30 nm–100 nm) which are released by exocytosis from multivesicular bodies of the endosome [21]. Microvesicles and exosomes have different biological functions [22], cargoes and modes of production. Microvesicles can be stimulatory or inhibitory depending on whether they are generated early or late during an inflammatory response [23]. Late response MV may include apoptotic or necrotic material, the former immunosuppressive, the latter immunostimulatory [23]. Exosomes may also be immunosuppressive, amongst other functions [22]. As discussed above, there is evidence that STBM can be both immunostimulatory and immunosuppressive [9], [10], [18], possibly reflecting a mixture of different vesicle types.

For experimental purposes, STBM can be produced *ex vivo* using several methodologies, some of which are more representative of *in vivo* STBM than others. Historically, “mechanically” derived STBM (mSTBM), which as the name suggests, are produced from mechanically disrupted villous tissue were used [24]. These are highly disruptive to endothelial cell monolayers [13] and inhibit endothelial cell and lymphocyte proliferation [25], but have limited proinflammatory activity [10]–[12]. More recently, STBM prepared from perfused placental lobules (pSTBM), which exhibit both anti-endothelial and proinflammatory activity, have been used [7], [10], [12], and are thought to be more representative of *in vivo* derived STBM [12].

The aim of this study was to characterise STBM produced from normal and PE affected placentas by these two methodologies, mechanical disruption and placental lobe dual perfusion and determine whether there were differences between those derived from normal and PE placentas which might explain their different functional properties. To do this we have developed a multicolour flow cytometry technique which enables us to accurately define STBM populations and the antigens they express. In particular we have investigated the expression of two anti-angiogenic molecules, fms-like tyrosine kinase 1 (Flt-1) and endoglin, both of which have soluble forms significantly elevated in the maternal circulation in PE and believed to play a role in the disorder. Western blotting for these molecules has been carried out in parallel. Biological activity of STBM associated Flt-1/soluble Flt-1 (sFlt-1) and endoglin was demonstrated by assessment of the ability of mSTBM to bind the ligands VEGF (vascular endothelial growth factor), PlGF (placental growth factor) and TGFβ (transforming growth factor β), required for maintenance of normal vascular endothelial function [26], as well as the effects of these ligands on endothelial cell disruption by mSTBM treatment *in vitro*. We also sought to determine the size distribution of vesicles from mSTBM and pSTBM preparations and whether there were differences between those prepared from normal and PE placentas. It was not possible to do this using flow cytometry as current digital instruments can only detect vesicles down to approximately 300 nm in size and are therefore not sufficiently sensitive to detect exosomes [27]. We have therefore utilized a new technology, Nanoparticle Tracking Analysis (NTA) which can measure cellular vesicles down to approximately 50 nm in size which includes exosomes [27]. Finally, using Western blotting (WB), the presence of exosome markers was investigated in normal and PE pregnancy placenta derived mSTBM and pSTBM preparations.

## Materials and Methods

### Ethics Statement

The Central Oxford Research Ethics Committee C approved this study and informed written consent was obtained from all recruited individuals.

### Tissue

Term human placentae (normal n = 22, PE n = 11) were obtained from the delivery suite at the John Radcliffe Hospital, Oxford from non-labouring healthy women with normal uncomplicated pregnancies and PE women, delivered by elective caesarean section. Only placentae collected within 10 min of delivery were used. Umbilical cords were obtained, with consent, from uncomplicated normotensive term pregnancies delivered by cesarean section (n = 5). Normal pregnant women were selected if they had no history of hypertension or chronic illness, a singleton pregnancy without known fetal abnormality, and natural conception. PE was defined as new hypertension (blood pressure ≥140/90 mm Hg on two consecutive occasions) and new proteinuria (24 h secretion of ≥500 mg), in the absence of urinary tract infection.

### Production of STBM

#### STBM produced by mechanical disruption (mSTBM)

Mechanically derived STBM (mSTBM) were prepared by a previously published method [24] with modifications outlined elsewhere [13]. In the present study, all buffers were passed through a 0.1 µM filter to minimalize the interference of background microparticles. Placentas were obtained from healthy (n = 13) and PE (n = 4) women and were processed immediately. Briefly, placenta tissue was scraped from villi and washed in ice cold 100 mM CaCl_2_ then PBS before being stirred in 0.9% NaCl buffer for 1 hour at 4°C. Cell debris was removed by centrifugation in a Beckman J6-M centrifuge at 600 g for 10 min at 4°C, then the supernatant was centrifuged at 150,000×g for 45 min at 4°C in a Beckman L8-80M ultracentrifuge. The resultant pellets were pooled and washed in sterile 0.1 µM filtered phosphate buffered saline (fPBS) before finally being resuspended in fPBS. Protein content was determined using a BCA protein assay kit and aliquoted samples stored at −80°C for subsequent analysis. Typical mSTBM yields were in the range of 50–100 mg total protein.

#### STBM produced by placental perfusion (pSTBM)

Perfusion derived STBM (pSTBM) were prepared using a dual placental perfusion system [28] modified as previously described [10]. Placentas obtained at cesarean section without labour, from healthy (n = 9) and pre-eclamptic (n = 7) women were processed immediately and, following an equilibration period, were perfused for 3 hr in a closed circuit. All perfusion media were passed through 0.1 µM filters to minimalize background microparticles that would interfere in subsequent microvesicle analysis. At the end of the 3 hr perfusion period, the maternal-side perfusate was centrifuged in a Beckman J6-M centrifuge at 600 g for 10 min at 4°C to remove large debris. The supernatant was centrifuged at 150,000×g (maximum) for 1 hr at 4°C in a Beckman L8-80 M ultracentrifuge to pellet the vesicles. The resultant pellets were pooled and washed in fPBS before finally being resuspended in fPBS to give a final protein content of 5 mg/ml, as assessed using a BCA protein assay kit, and stored in aliquots at −80°C until subsequent use. Typical pSTBM yields were in the range of 25–50 mg total protein.

### Flow Cytometric Analysis of STBM

#### Flow cytometer set up and determination of limit of detection

Analysis of mSTBM and pSTBM was carried out by multicolour flow cytometry, using a BD LSRII Flow Cytometer (BD Biosciences, Oxford UK) equipped with a 488 nm (blue) and 633 nm (red) laser. All data were analysed using FACS DIVA software (Becton Dickinson, Oxford, UK). Firstly, the limits of detection of the flow cytometer were established using standard size calibration beads. This is a common approach that has been used in many studies to set the size gate for microvesicle (MV) analysis [29]–[32]. 1 µm Fluoresbrite YG microspheres (Polysciences Europe GmbH, Eppelheim, Germany) and NIST polystyrene YG microspheres; 200 nm, 290 nm, 390 nm, 590 nm (Thermo Scientific, Fremont, CA) were diluted accordingly in fPBS and used to determine the optimum side scatter and forward scatter voltages for MV detection with minimum interference from background machine noise [27]. 1 µm Fluoresbrite YG microspheres were then used to set a 1 µm limit gate to exclude larger debris and contaminating platelets from the analysis. Events that fell into this gate were then classified as being ≤1 µm in size. Logarithmic voltages were used for all channels. As STBM sample events were collected over 2 mins, BD Trucount tubes were run to monitor flow rate stability. Prior to running STBM samples, fPBS was also analysed in triplicate for 2 mins to assess the level of background contaminating events.

#### Conjugated dyes and antibodies used for STBM multicolour flow cytometry

The following membrane stain and fluorescence conjugated antibodies were used for STBM phenotyping. All of the antibodies and corresponding IgG controls were mouse monoclonals and purchased directly conjugated to the appropriate fluorochrome, with the exception of the in-house NDOG2 monoclonal antibody and commercially purchased unconjugated IgG1 isotype control antibody. Bio-maleimide (Mal) conjugated to either Alexa 680 or Alexa 488 (Invitrogen) was used as a general MV marker as it binds to sulphydryl groups present in proteins. The NDOG2 antibody and its IgG1 control were conjugated to either RPhycoerythrin (RPE) or fluorescein isothiocyanate (FITC) using commercially available conjugation kits (Lightning Link; Innova Biosciences) according to the manufacturer’s instructions and was used to stain MV originating from the syncytiotrophoblast (STB). NDOG2 is a STB specific antibody that recognises placental alkaline phosphatase (PLAP) [33]. Non-STB MV in the preparations were identified using W6/32 conjugated to Alexa 647 (AbD Serotec) which binds to MHC class I, expressed by all cells in the body except STB and erythrocytes. Finally, the presence of the Flt-1/sFlt-1 and endoglin (Eng) on STBM was demonstrated with the binding of anti-Flt-1 mouse MAb conjugated to allophycocyanin (APC) (R&D Systems) and anti-endoglin mouse MAb conjugated to RPE (R&D Systems). Prior to use, all antibodies and bio-maleimide were filtered through Nanosep 0.2 µm centrifugal devices (Pall Life Sciences) to minimalize interference by background microparticles. All antibodies and bio-maleimide were also titrated to ensure their use at the optimum concentration. Fluorochrome compensation for multicolour STBM flow cytometry was set-up using BD CompBeads (BD Biosciences) labelled with fluorescence conjugated antibodies and fluorescence bio-maleimide labelled STBM.

#### STBM sample preparation for multicolour flow cytometry

Frozen aliquots of mSTBM and pSTBM were thawed at 37°C. Prior to analysis, the vesicle content of each sample was measured using the flow cytometer, to calculate the sample volume required for a final event rate of ∼40,000 events/2 mins. This step was carried out to ensure a consistent STBM:antibody ratio across samples. All samples were then blocked with 0.2 µm filtered Fc receptor blocker (10 µL; Miltenyi) for 10 min at 4°C before the addition of either fPBS (unlabeled control), fluorescence conjugated IgG controls (conjugated IgG negative control), or fluorescence conjugated Mal and fluorescence conjugated antibody (labeled STBM). Due to the lack of a negative control for Mal, a FMO (fluorescence minus one) tube was set up for each sample, that contained fluorescence conjugated antibodies alone (Mal FMO control) (Roederer 2002). Tubes were incubated for 15 min at R/T in the dark. Stained samples were then made up to 200 µL with fPBS and acquired immediately for 2 min on the flow cytometer (Becton Dickinson LSR II). Gates were firstly set so that ≤1% of cells stained positive in the appropriate negative controls. The cells labelled with the antibodies of interest were then compared with the negative control using Diva flow cytometry software (Becton Dickinson).

#### Flow cytometric determination of the purity of STBM preparations

To assess the proportion of STBM and non-STB derived MV contained in the four STBM preparation types (normal and PE pSTBM and normal and PE mSTBM) a pooled sample was generated for each, then appropriately diluted, blocked with Fc receptor blocker and labelled with Mal-Alexa 488, NDOG2-RPE and W6/32-Alexa 647 in parallel with appropriate controls. The flow cytometer was set up as outlined above and all sample events collected over 2 min.

#### Four colour flow cytometric analysis

Four colour flow cytometry was used to identify STBM and their associated antigens. Individual pSTBM and mSTBM preparations from normal and PE placentas were stained with the following reagents: Mal-Alexa 680; NDOG2-FITC, anti Flt-1-APC and anti Eng-RPE. Unlabeled, FMO control (containing NDOG2-FITC, anti Flt-1-APC and anti endoglin-PE) and IgG negative control tubes were also set up for each sample. All samples were run as previously described.

### STBM SDS PAGE and Western Blotting

For analysis of PLAP, Flt-1, endoglin, the exosome markers Lamp I, Alix, CD63, CD9, TSG101 and actin, pSTBM and mSTBM were lysed (on ice, 30 min) in SDS-PAGE sample buffer containing protease inhibitor cocktail (Roche Diagnostics). Lysed samples were then diluted in reducing (PLAP, Flt-1, endoglin, Lamp I, Alix, CD9, TSG101 and actin) or non-reducing (CD63) sample buffer to give a final protein concentration of 10 µg/30 µL (PLAP, Flt-1, endoglin and actin) or 20 µg/30 µL (Lamp I, Alix, CD63, CD9, TSG101 and actin). Samples were then boiled and centrifuged (13,000×g for 10 min) prior to separation by SDS/PAGE (Invitrogen) and semi-dry transfer to PVDF membrane (Biorad). Non-specific binding was blocked with TBS-T (20 mM Tris/HCl, 137 mM NaCl, 0.1% Tween-20, pH 7.6) containing 5% BLOTTO (Santa Cruz Biotechnology Inc.). Membranes were incubated O/N with antibodies against PLAP (NDOG2), Flt-1 (Abcam), endoglin (G4484 mouse MAb gift from Prof Letarte, University of Toronto), Lamp I (BD Transduction Laboratories), Alix (New England Biolabs), CD63 (Abcam), CD9 (Abcam), TSG101 (Abcam) or actin (Abcam) at 4°C, and then washed in TBS-T, before incubation with the appropriate horseradish-peroxidase-conjugated secondary antibody (Dako). All antibodies were diluted in blocking buffer. After washing, blots were treated with an enhanced chemiluminescence system (Pierce) and exposed to Hyperfilm ECL (GE Health Care). The resultant band densities were quantified using Image J software [Abramoff MD, Magelhaes PJ, Ram SJ. Image Processing with Image. J Biophotonics Int 2004;11∶36e42], available from http://rsb.info.nih.gov/ij/and actin densities used to normalize data.

### mSTBM Binding of VEGF, PlGF and TGFβ

To investigate the binding of VEGF_121_, VEGF_165_, PlGF and TGFβ to normal mSTBM a pool of 13 preparations were double diluted in dilution buffer (PBS containing 4 mM EDTA, 0.1% BSA and 0.001% tween 20 at pH 7.4). 1 ng/ml of either VEGF_121_, VEGF_165_, PlGF, TGFβ (R&D Systems) or vehicle control was then added, the tubes vortexed and incubated at 4°C overnight with gentle mixing. Tubes containing the equivalent volume of dilution buffer plus 1 ng/ml VEGF_121_, VEGF_165_, PlGF or TGFβ but without mSTBM were set up in parallel to control for any non-STBM related losses. All binding experiment tubes were set up in triplicate. At the end of the incubation period the tubes were ultracentrifuged to remove mSTBM (150,000×g for 1 hr at 4°C in a Beckman L8-80M ultracentrifuge) and the supernatant assayed by ELISA (R&D Systems) to determine the recovery of VEGF_121_, VEGF_165_, PlGF and TGFβ relative to the no STBM control.

### Effect of VEGF, PlGF and TGFβ on STBM Disruption of Endothelial Cells in Culture

The effect of VEGF_121_, VEGF_165_, PlGF or TGFβ treatment on the disruption of endothelial cell monolayers by mSTBM treatment was tested. Human umbilical vein endothelial cells (HUVEC) were isolated and maintained as described previously [34]. Cells between passages 4 and 5 were used for all experiments. On day 1, HUVEC (90,000 cells/well) were plated out into 1% gelatin coated 24-well plates in 500 µL HUVEC culture medium (CM) (M199 containing l-glutamine supplemented with 10% (vol/vol) heat-inactivated FCS, endothelial cell growth supplement (ECGS; 30 µg/ml), heparin (90 µg/ml), and penicillin, streptomycin, and neomycin (50 U/ml, 50 µg, and 100 µg/ml, respectively). The cells were left for 48 h at 37°C in 5% CO2 and 95% air to allow cells to grow to approximately 80–90% confluence. On day 3, CM was removed and cells washed in basic CM (M199 containing l-glutamine supplemented with 10% (vol/vol) heat-inactivated FCS and penicillin, streptomycin, and neomycin (50 U/ml, 50 µg, and 100 µg/ml, respectively)) before treatments, made up in basic CM, were added for 16 h. mSTBM (pool of 13 preparations from normal placentas) were tested at 75 µg/mL and VEGF_121_, VEGF_165_, PlGF and TGFβ tested at 100 ng/mL. At the end of the treatment period the cells were washed with Hanks balanced salt solution and stained with CMFDA (1 µM) for 1 h in serum free CM (M199 containing l-glutamine and penicillin, streptomycin, and neomycin (50 U/ml, 50 µg, and 100 µg/ml, respectively)), before being washed with HUVEC CM and images taken using a Leica DMIRE2 inverted fluorescence microscope, Hamamatsu Orca camera and Simple PCI software. The resultant images were analysed using Image J software, to obtain the percentage HUVEC coverage by measuring the number of pixels above a constant background threshold.

### Nanoparticle Tracking Analysis of STBM Preparations

Size distribution profiles of mSTBM and pSTBM from normal and pre-eclampsia placentas were obtained using Nanoparticle Tracking Analysis (NTA) as described by Dragovic et al. (2011), with minor modifications. In this system vesicles are visualised by light scattering using a light microscope. A video is taken using a high sensitivity camera and the NTA software tracks the Brownian motion of individual vesicles and calculates their size and concentration. All samples were analysed using an NS500 instrument (Nanosight Ltd, Amesbury, UK). Each sample was diluted in PBS to give approximately 5×10^8^ vesicles/mL. The samples were automatically introduced into the sample chamber and ten video recordings of 20 seconds in duration were captured at 30 second intervals on different aliquots of the sample. Shutter speed and gain were optimised for biological vesicles in the 80–250 nm size range. NTA post acquisition settings were also optimised and kept constant between samples and each video was then analysed to give the mean, mode and median vesicle size.

### Nanoparticle Tracking Analysis of SH-SY5Y Exosomes

Neuroblastoma cell line SH-SY5Y exosomes were isolated and their size distribution profile obtained using NTA as previously described [35].

### Statistical Analysis

Mann Whitney non parametric test was used for all comparisons. Statistical analyses were carried out using Prism software. Values of p<0.05 were considered to be statistically significant.

## Results

### Patient Data

Demographic and clinical characteristics of the study participants are shown in Table 1. There was no significant difference in parity (not shown) or maternal age at the time of booking. Body mass index and maximum diastolic and systolic blood pressures were all significantly higher in the PE patients compared to the normal pregnant study participants (Table 1). As would be expected, gestational age and birth weight were both significantly lower in PE affected pregnancies (Table 1).

**Table 1:** Patient details for normal pregnant (n = 22) and preeclamptic women (n = 11).

	Perfusion	Perfusion	Mechanical		Mechanical
	Normal Pregnant (n = 9)	PE (n = 7)	Normal Pregnant (n = 13)	PE (n = 4)	
**Age (yrs)**	33 (26–38)	39 (24–44)	35 (25–43)	35 (28–39)	
**Gestation (Wks)**	39 (38^+1^–42)	35^+3^ (30–39^+1^)*	39 (38^+3^–40^+4^)	37.5 (32^+6^–39)*	
**BMI**	24.4 (17.6–42.5)	26.4 (22.3–39.1)**	26.1 (19.4–36.1)	27.8 (25–39)**	
**Maximum Systolic BP (mmHg)**	120 (105–137)	170 (150–205)***	120 (100–150)	159 (150–170)***	
**Maximum Diastolic BP (mmHg)**	74 (40–83)	105 (96–155)***	70 (30–80)	107.5 (90–118)***	
**Proteinuria (mg/24** **h)**	NAD	1167 (660–5831)	NAD	1027.9 (612–3481)	
**Birthweight (g)**	3430 (2925–4156)	2268 (1200–3500)**	3787 (3155–4300)	2788 (1460–3417)**	

Values expressed as median (range). *P<0.003; **P<0.001; ***P<0.0003. P values are results of Mann Whitney test comparison of normal vs PE within each syncytiotrophoblast microvesicle preparation group. (NAD; nothing abnormal detected).

### Four Colour Flow Cytometric Analysis

#### Flow cytometer detection limit and set up

**Figure 1:**
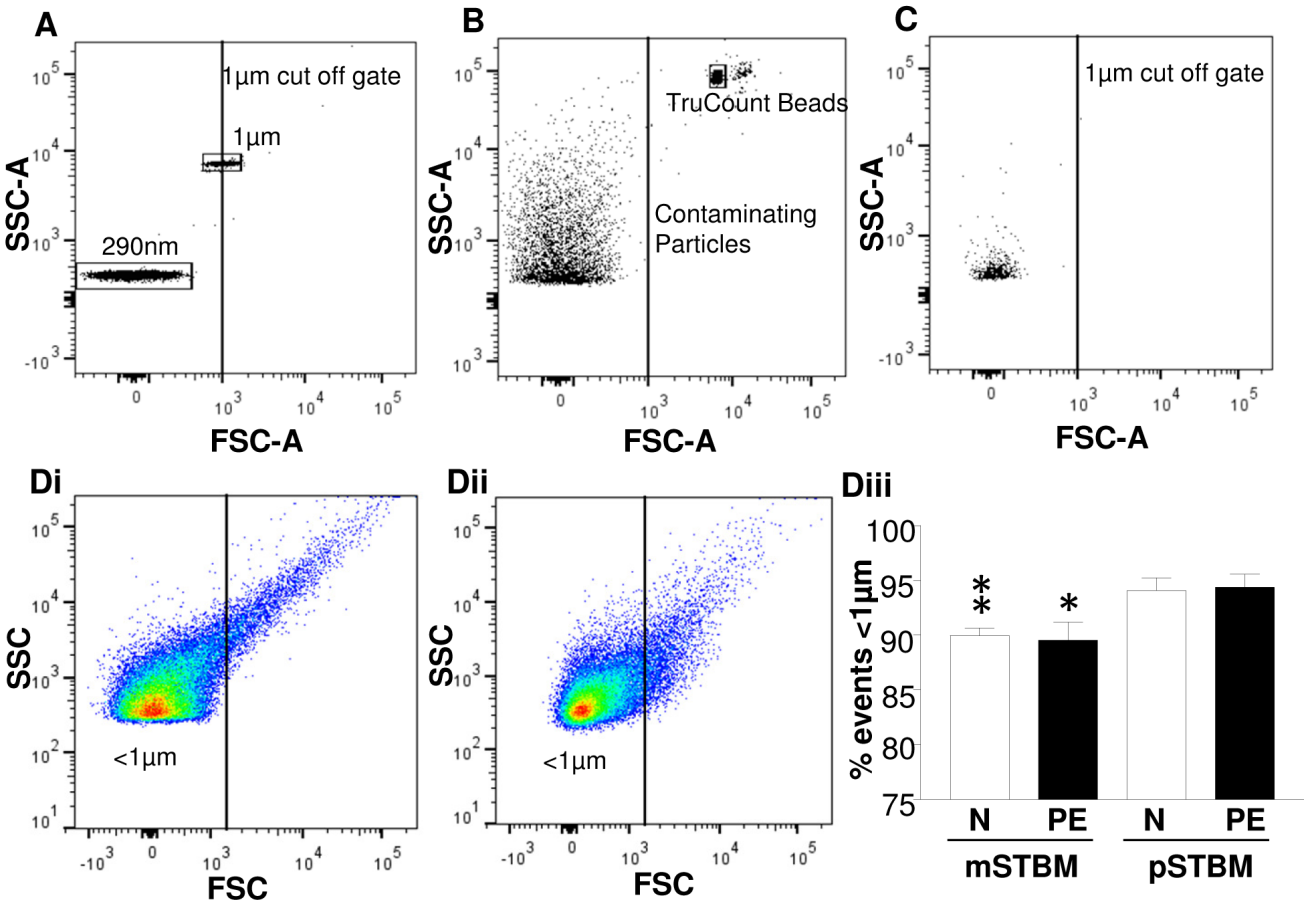
LSRII Flow Cytometer set up. A) SSC & FSC voltages were adjusted to visualise 290 nm and 1 µm microspheres to establish the microvesicle analysis gate. B) Trucount beads were analysed for two minutes demonstrating large numbers of background particles present in the Trucount tube. C) Two minute analysis of 0.1 µm filtered PBS showing the low number of background events present. D) FSC vs. SSC density plots with ≤1 µm gate of representative i) mSTBM and ii) pSTBM preparations and iii) barchart showing a significantly lower percentage of events ≤1 µm in both normal and PE mSTBM compared to pSTBM. *P<0.05 and **P<0.01.

We have previously shown using a range of fluorescent beads (200 nm, 290 nm, 390 nm, 590 nm and 1 µm) analysed on forward scatter (FSC) and side scatter (SSC) that the limit of sensitivity of our BD LSRII flow cytometer is approximately 300 nm [27]. A gate was therefore set to include beads of only 290 nm –1 µm (Figure 1A) to minimise background noise. As the BD Trucount tubes used to determine the number of vesicles present contain many contaminating particles of a size which fall within the microvesicle gate (Figure 1B) the beads were run separately rather than with the samples. The flow cytometer flow rate was shown to be stable (typical inter-assay CV of 3% and intra-assay CV of 4% over a one month period), while background contaminating events were kept to a minimum (typical levels of 2.5% were obtained) with regular cleaning of the flow cytometer (Figure 1C). FSC vs SSC profiles clearly differed between pSTBM (Figure 1Di) and mSTBM (Figure 1Dii), with mSTBM preparations from both normal and PE placentas containing significantly fewer events <1 µm in diameter (P<0.01)(Figure 1Diii).

#### Microvesicle gating strategy

**Figure 2:**
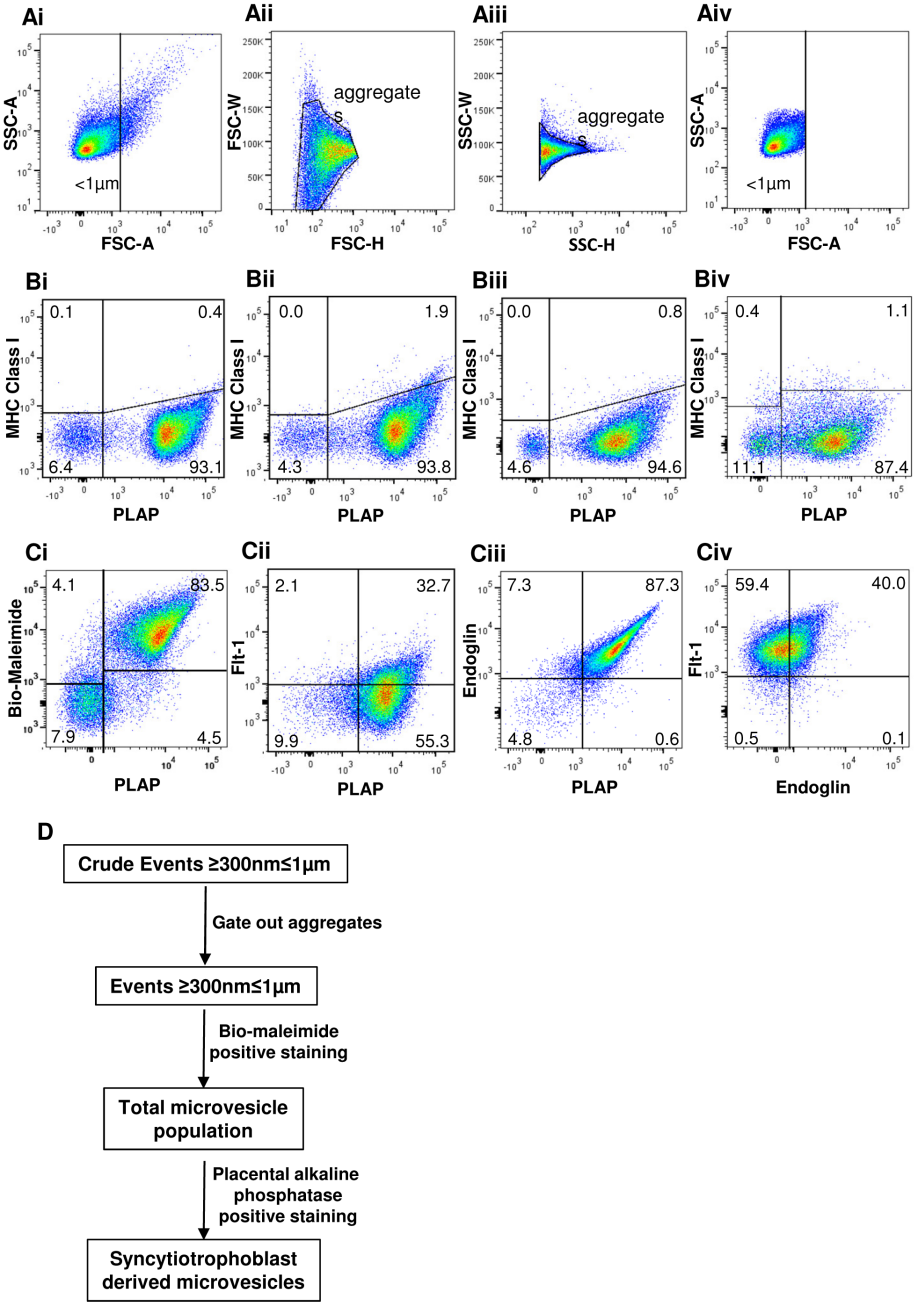
Four colour flow cytometric analysis of placental microvesicles. Representative results for pSTBM analysis are shown in A(i-iv) and C(i-iv). Phenotyping of MV was carried out as follows: (Ai) Crude events >290 nm<1 µM were identified by FSC vs. SSC. (Aii) Aggregates were removed from the analysis using FSC-Height Vs FSC-Width, (Aiii) followed by SSC-Height Vs SSC-Width plots to resolve events >290 nm<1 µM. (Aiv) The total MV population was identified as bio-maleimide positive within this gate and this population was then used in subsequent analyses of MV associated ligands. pSTBM and mSTBM preparation purity was assessed using quadrant plots of NDOG2 (anti-placental alkaline phosphatase (PLAP) antibody) vs W6/32 (anti-MHC class I antibody) displaying the total MV population to identify syncytiotrophoblast derived MV (STBM) and other contaminating MV in (Bi, iii) normal pregnancy and (Bii, iv) preeclampsia (Bi, ii) mechanical and (Biii, iv) perfusion derived MV preparations respectively. The expression of Flt-1 and endoglin were investigated as follows: (Ci) Total MV and STBM populations were identified using bio-maleimide-Alexa 680 (Mal) staining and Mal with NDOG2-FITC double staining, respectively. Flt-1 or endoglin positive placental MV were identified by displaying the total MV population on either a (Cii) Flt-1-APC Vs NDOG2-FITC or (Ciii) endoglin-PE Vs NDOG2-FITC quadrant plot. Finally, (Civ) endoglin/Flt-1 double positive placental MV were identified by plotting Mal/NDOG2 double positive MV onto an endoglin-PE Vs Flt-1-APC quadrant plot. (D) Flow chart to describe the flow cytometric identification of syncytiotrophoblast derived microvesicles.

Phenotyping of MV was carried out as follows: Crude events ≥300 nm≤1 µm were identified on a forward scatter (FSC) vs. side scatter (SSC) plot (Fig. 2Ai). Aggregates were removed from the analysis by displaying crude events ≥300 nm≤1 µm on a FSC-Height Vs FSC-Width plot and gating around the main population (Fig. 2Aii), the results of which were then displayed on a SSC-Height Vs SSC-Width plot (Fig. 2Aiii) and a gate again placed around the main population. All fluorescence staining was then analysed on this population of events ≥300 nm≤1 µm [32].

#### Flow cytometric determination of the purity of STBM preparations

The purity of the STBM preparations was determined using three colour flow cytometry. The total MV population was identified as being the events ≥300 nm≤1 µm positive for Mal-Alexa 488 (Figure 2Aiv). The total MV population was then displayed on PLAP vs W6/32 quadrant plots to identify STBM (PLAP +ve W6/32–ve) and non-STBM (PLAP –ve W6/32+ve) respectively (Figure 2B). No difference was seen between normal and PE STBM and non-STBM content of mSTBM (93.1% vs 93.8% and 0.1% vs 0% respectively) (Figure 2Bi and ii). Pooled pSTBM preparations however showed a difference in purity when prepared from normal or PE placentas. The PLAP +ve vesicle content was higher in pSTBM preparations from normal (94.6%) compared to PE (87.4%) placentas (Figure 2Biii and iv). This was reflected predominantly by an increase in the percentage of W6/32 and NDOG2 negative MV, with 4.6% in normal compared to 11.1% in PE pSTBM preparations (Figure 2Biii and iv).

#### Four colour flow cytometric analysis of STBM phenotype

To determine whether mSTBM and pSTBM expressed Flt-1 or endoglin, events ≥300 nm≤1 µm were displayed on a Mal vs PLAP quadrant plot to determine the Mal and PLAP double positive MV (STBM) population (Figure 2Ci). To determine the STBM positive for Flt-1 or endoglin, the total MV population was then displayed on quadrant plots of Flt-1 vs PLAP (Figure 2Cii) and Eng vs PLAP (Figure 2Ciii). Finally, to determine the percentage STBM positive for both Flt-1 and endoglin, a quadrant plot of Eng vs Flt-1 was drawn showing the STBM population (Figure 2Civ).

### Effect of Preparation Methodology and Preeclampsia on Surface Antigen Expression of STBM

**Figure 3:**
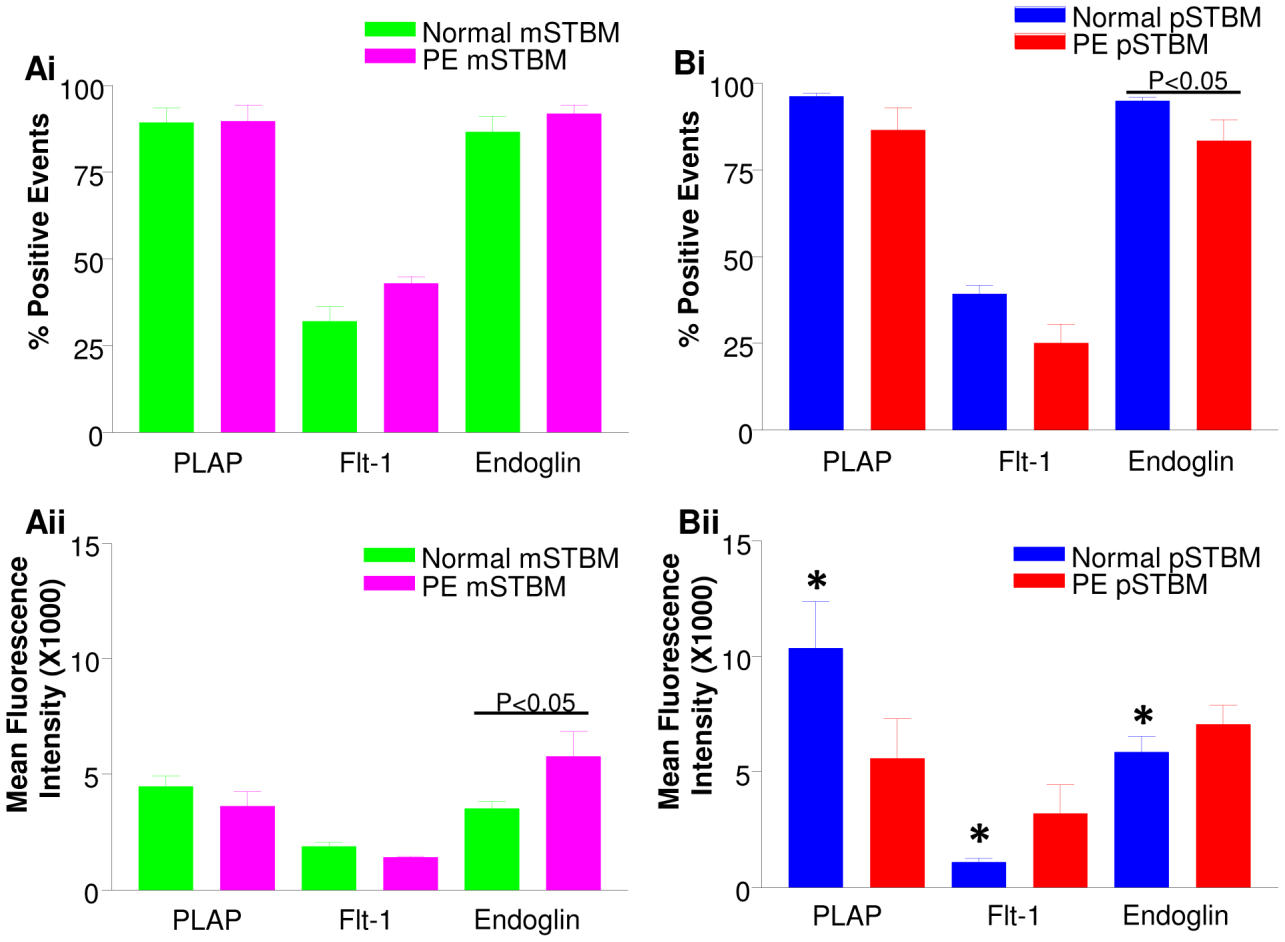
Bar charts showing results of multi-colour flow cytometry analysis of (A) mechanically derived (mSTBM) and (B) perfusion derived (pSTBM) placental vesicle preparations. (i) Percentage positive events and (ii) mean fluorescence intensity of placental alkaline phosphatase (PLAP), Flt-1 (vascular endothelial cell growth factor receptor-1) and endoglin stained total microvesicle (MV) population (for PLAP) and STB derived MV (for Flt-1 and endoglin). p values within a barchart are a comparison between PE and normal whereas * denotes a comparison between mSTBM and pSTBM. *P<0.05 compared with normal mSTBM.

Analysis by flow cytometry revealed that placental MV preparations, produced by either perfusion or mechanical disruption and from normal or PE placentas, all contained similarly high proportions of MV positive for PLAP, confirming their STB origin (Figure 3Ai and Bi). Likewise, preparation method and PE did not significantly affect the percentage of mSTBM or pSTBM positive for Flt-1 (Figure 3Ai and Bi). Unlike preparation method, PE significantly decreased the percentage of pSTBM but not mSTBM positive for endoglin (P<0.05)(Figure 3Bi). However, there were no significant differences between preparation methods or PE in the percentage of STBM expressing both Flt-1 and endoglin (mSTBM norm 31.9±4.3% and PE 42.9±2.0; pSTBM norm 40.0±2.4% and PE 28.8±6.0%). All Flt-1 positive STBM were double positive for endoglin (Figure 2Civ).

The mean fluorescence intensity (MFI) for each antibody was also investigated. The MFI indicates the amount of antigen carried on each MV positive for that antigen. PLAP MFI was significantly lower on normal mSTBM compared to pSTBM (P<0.05) (Figure 3Aii and Bii). Expression analysis of Flt-1 and endoglin on STBM showed several differences between the two methodologies used, with Flt-1 expression significantly lower and endoglin significantly higher on normal pSTBM compared to mSTBM (P<0.05) (Figure 3Aii and Bii). PE tended to decrease PLAP expression on pSTBM, although this did not reach significance, and significantly increased endoglin expression on mSTBM only (P<0.05) (Figure 3Aii).

### SDS PAGE and Western Blotting of STBM Preparations

**Figure 4:**
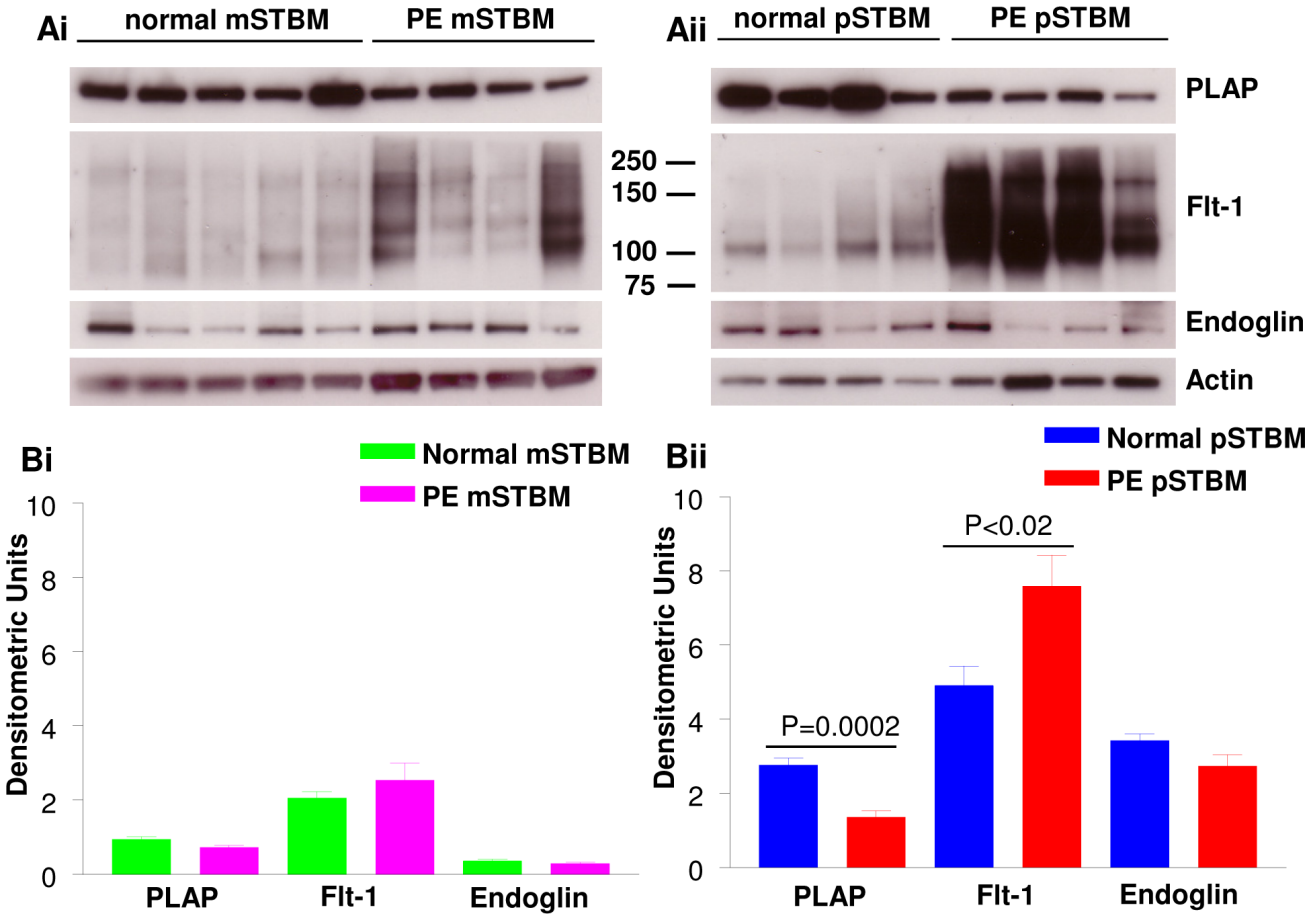
Western blotting analysis of placental vesicle preparations. A) Representative immunoblot images and B) the corresponding densitometric analysis of placental alkaline phosphatase (PLAP), VEGF receptor-1 (Flt-1) and endoglin in syncytiotrophoblast microvesicles (STBM) prepared by i) mechanical disruption (mSTBM) and ii) placental perfusion (pSTBM) of normal and preeclampsia (PE) affected pregnancies. P values show the comparison between normal and PE derived pSTBM.

Semi-quantitative WB analysis of mSTBM and pSTBM was carried out to validate expression patterns seen with PLAP, Flt-1 and endoglin by flow cytometry and further investigate the form(s) of Eng and Flt-1/sFlt-1 protein present in normal and PE mSTBM and pSTBM. It is important to note that flow cytometry was carried out on non-permeabilised vesicles which only measures surface protein expression, whereas WB measures total (both surface and intravesicular) proteins. For normal and PE mSTBM, results of WB analysis showed no significant difference in expression of PLAP, Flt-1 or endoglin (Fig. 4Ai and ii). However, for pSTBM, PLAP levels were significantly lower on PE derived STBM compared to STBM isolated from normal pregnancy placentas (Fig. 4Bi and ii; P = 0.0002), a trend seen with flow cytometry. Western blotting analysis of Flt-1/sFlt-1 clearly showed multiple forms corresponding to 90–100 kDa, ∼115 kDa and 160–190 kDa present to varying degrees in all samples. In mSTBM (Fig. 4Aii) and in pSTBM (Fig. 4Ai), Flt-1/sFlt-1 isoforms tended to be more highly expressed in PE derived STBM compared to STBM isolated from normal pregnancy placentas. However, densitometric analysis showed significantly higher total Flt-1 isoform expression in PE pSTBM only (P<0.02; Fig4Bii) compared to those isolated from normal placentas.

### Syncytiotrophoblast Microvesicle Binding of VEGF, PlGF and TGFβ

**Figure 5:**
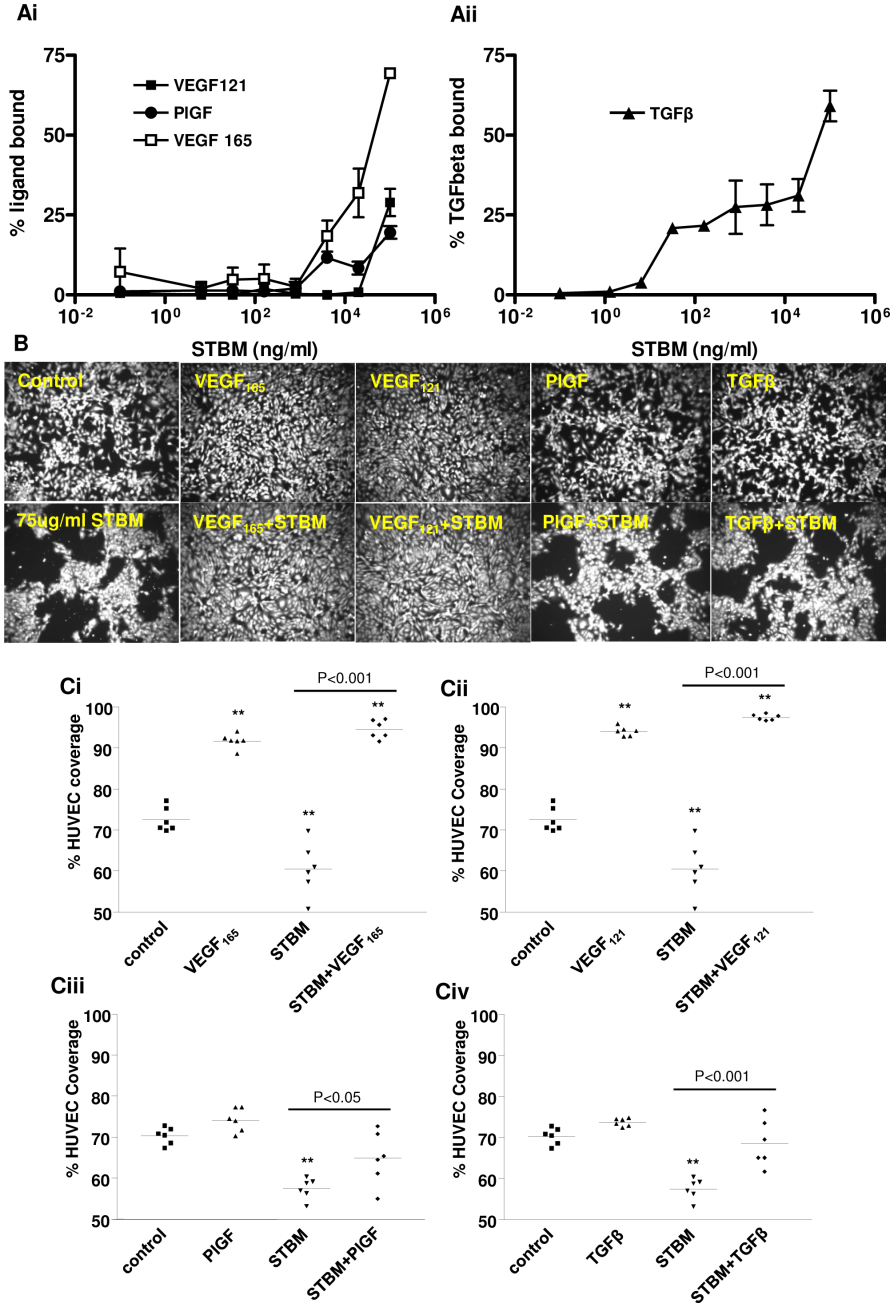
Interaction of placental vesicle preparations with angiogenic factors. Binding of Ai) VEGF_121_, VEGF_165_ and PlGF and Aii) TGFβ to increasing amounts of pooled mSTBM from 13 normal placentas. B) Representative fluorescence micrographs and C) the corresponding percentage coverage data for human umbilical vein endothelial cell monolayers treated with VEGF_121_, VEGF_165_, PlGF and TGFβ (100 ng/mL) in the presence and absence of normal mSTBM (75 µg/mL, pool of 13 preparations). Median values are indicated on the graphs. **P<0.001 compared with untreated control wells.

Having shown that Flt-1 and endoglin were present on STBM we then sought to determine whether they might have a functional role and bind ligands for Flt-1 (VEGF_121_, VEGF_165_, PlGF) and endoglin (TGFβ). These assays were only performed on a pool of normal mSTBM (n = 13) due to the availability of the material. The results showed that mSTBM bound all four growth factors (Figure 5 Ai and Aii). The use of VEGF_121_ (which lacks the heparin binding domain (HBD)) and VEGF_165_ (which contains HBD) in parallel enabled us to discriminate between VEGF binding to surface receptors or heparan sulphate. PlGF-1, like VEGF_121_, binds Flt-1 but does not contain a HBD. mSTBM bound PlGF and VEGF _121_ to a similar extent, but bound more VEGF_165_, suggesting that both receptors and heparan sulphate were responsible for binding (Fig. 5A). The capacity of mSTBM to bind TGFβ was much higher, as ∼100–1000 fold less mSTBM was required to bind 25% of the added TGFβ compared to VEGF and PlGF (Fig. 5B). As TGFβ also contains a HBD, binding to both endoglin and heparan sulphate was possible.

### Effects of VEGF, PlGF and TGFβ Treatment on Endothelial Cell Disruption by mSTBM

As mSTBM were able to bind VEGF_121_, VEGF_165_, PlGF and TGFβ, we wanted to investigate whether these Flt-1 and endoglin ligands could reduce HUVEC monolayer disruption by mSTBM *in vitro*. As shown previously [13] mSTBM significantly disrupted the HUVEC monolayer (P<0.001; Figure 5B and C). All of the ligands tested significantly blocked the disruptive effects of mSTBM, shown by increased HUVEC coverage in the presence of mSTBM and ligand compared to mSTBM treatment alone (VEGF_121_, VEGF_165_ and TGFβ P<0.001, PlGF P<0.05; Figure 5C). When treated with ligand alone, only VEGF_121_ and VEGF_165_ stimulated HUVEC proliferation, evident by increased HUVEC coverage compared to untreated controls (Figure 5Ci and 5Cii).

### Nanoparticle Tracking Analysis (NTA) of STBM and Exosome Marker Expression

**Figure 6:**
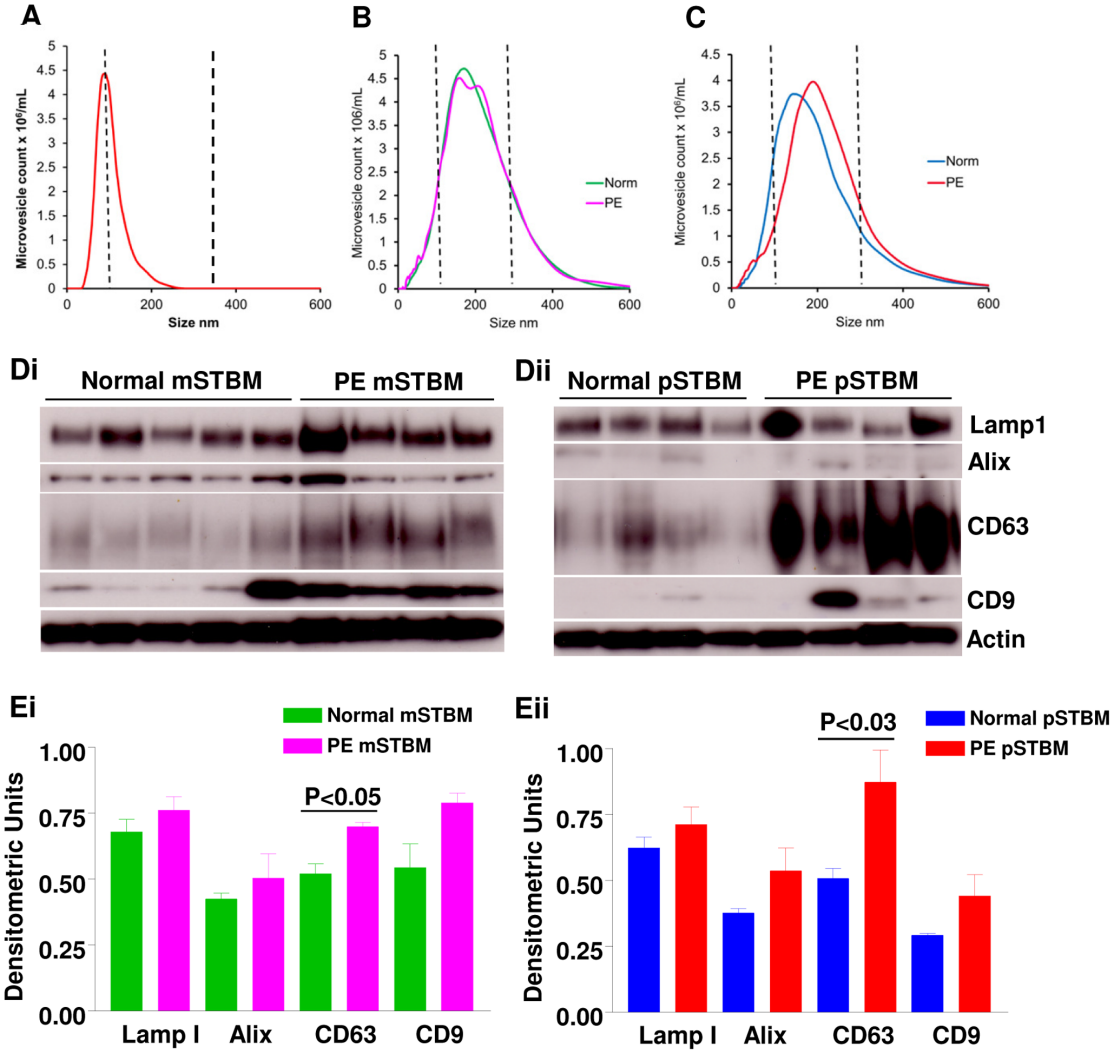
Analysis of vesicle size distribution and exosome marker expression of SH-SY5Y neuroblastoma cell line and placental vesicle preparations. Nanosight Tracking Analysis size distribution profiles for (A) a representative SH-SY5Y neuroblastoma cell line exosome preparation, (B) mechanically derived syncytiotrophoblast microvesicle preparations and (C) placental perfusion (pSTBM) from normal (norm) and preeclampsia. (D) Representative immunoblot images and (E) the corresponding densitometric analysis of the exosome markers Lamp I, Alix, CD63 and CD9 in i) mSTBM and ii) pSTBM. Densitometric values were normalized using actin as a loading control.

STBM were analysed using NTA to determine their size distribution, which relates to their composition in terms of exosomes and microvesicles. We first analysed a preparation of vesicles derived from the culture supernatant of neuroblastoma cell line SH-SY5Y. These were found to have a peak modal size of 93 nm, with a size range of 50–250 nm, consistent with that of exosomes (Fig. 6A). We next analysed 13 mSTBM preparations from normal placentas and 4 mSTBM from PE placentas where the modal peak size and size range were 140–235 nm and 50–500 nm respectively (Fig. 6B), suggesting that mSTBM preparations contain both exosomes and microvesicles. There was no difference in the size distribution profiles between the mSTBM from normal and PE placentas. pSTBM had a similar size distribution, with vesicles ranging from 50–500 nm and modal peak size between 111–211 nm (Fig. 6C). However, PE derived pSTBM (n = 7) showed a significant increase in both median (P = 0.004) and modal size (P<0.001; Fig. 6C) compared to normal placenta pSTBM (n = 9) (201 nm vs 166 nm and 191 nm vs 149 nm respectively). This suggests that pSTBM from PE placentas contain fewer vesicles in the exosome size range and more in the microvesicle range, which may reflect different patterns of shedding in the disease state. It is also clear from NTA that both pSTBM and mSTBM contain a large number of vesicles (>75%) undetectable by flowcytometry (ie. smaller than ∼300 nm) (Fig. 6).

Western blotting of mSTBM and pSTBM was carried out for Lamp1, Alix, CD63, CD9 and TSG101 to confirm the presence of exosomes. All of the exosome markers except TSG101 were detectable at varying levels (Fig. 6D), although were no significant differences between the types of vesicles (mSTBM v pSTBM). Only CD63 showed a significant elevation on both mSTBM and pSTBM from pre-eclampsia placentas compared to normal (P<0.05 and P<0.03 respectively)(Fig. 6E).

### Conclusions

Better characterization of placental vesicles is required to understand their role in both physiological and pathological pregnancies. The present study set out to address this issue. By developing a multi colour flow cytometric methodology for use with STBM preparations, we were able to investigate purity of placental MV preparations and the presence of PE related antigens on STBM from placentas of normal healthy pregnancies and those affected by PE, prepared using two methods. Employing NTA technology also allowed the investigation of the effects of preparation method and PE on size distribution of ex vivo prepared placental vesicles. The two techniques complement each other with flow cytometry detecting and phenotyping the larger MV (∼300 nm−1 µm) and the superior sensitivity of NTA allowing analysis of exosomes and smaller MV (∼50–400 nm). We have confirmed, in parallel, the presence of exosomes in the different STBM preparations by western blotting for common exosome markers (LAMP1, Alix, CD63 and CD9).

Multicolour flow cytometry has enabled us to accurately assess the purity of the placental MV preparations. When analysing MV by flow cytometry every precaution needs to be taken to reduce the background event count, such as filtration of buffers and regular cleaning of the machine. By initially using bio-maleimide staining to identify the total MV population, we were able to further minimize the contribution of non-cellular derived particles to the overall analysis. By triple labeling samples with bio-maleimide and NDOG2 and W6/32 antibodies the percentage of the total MV population derived from STB produced by perfusion was shown to be consistently high, in agreement with those reported by Guller et al (2011) of 93–95%. No major effect of preparation method or PE on the levels of PLAP positive MV was seen, apart from a modest decrease in PLAP positive pSTBM derived from PE placentas. Decreased PLAP expression, rather than an increase in the proportion of contaminating vesicles was confirmed by a significant decrease in PLAP MFI and by a significant decrease in pSTBM PLAP expression using western blotting. This suggests that a difference in STBM content does not account for the previously reported variation in biological activity of mSTBM and pSTBM [14].

During placental perfusion, STBM release occurs in conditions mimicking the *in vivo* environment ie. the architecture of the placental tissue is conserved and the integrity of the STB layer maintained, unlike mechanical disruption where extensive damage of the villous tissue occurs. Likewise, PE is associated with placental disruption, shown by abnormal STB turnover, with increased trophoblast apoptosis [36] and necrosis, resulting in increased release of placental debris into the maternal circulation [5], [7], suggesting that PE placentas are compromised prior to the ex vivo production of STBM, damage that could alter the mechanism of release, size and phenotype of MV. Mechanical disruption increased the proportion of vesicles >1 µm, measured by flow cytometry, suggestive of an increase in apoptotic and necrotic vesicles [21]. As polystyrene beads have a higher refractive index then extracellular vesicles [37] the true size of the vesicles detected by flow cytometry could be much larger, as such, an effect of PE also on vesicles >1 µm cannot be ruled out. PE and mechanical disruption both increased the mean and modal size of vesicles detectable by NTA, suggesting that release of vesicles in the smaller size range is also affected. NTA analysis of pSTBM showing a shift in MV size profile with PE suggests an imbalance between exosome and MV release and a shift towards increased proinflammatory activity. This supports the results of Holder et al 2012, with electron microscopic analysis suggesting the release of larger and more disrupted MV with greater inflammatory activity from cultured PE placental explants compared to those from normal pregnancy. Increased STB apoptosis may also decrease exosome production as reported for tumour cells in culture [38]. This raises the question of whether reduced exosome production and/or increased large MV release ex vivo reflects the in vivo situation and whether this occurs during the early stages of pregnancy when immune modulation is critical to placental development [20], [39].

By using multicolour flow cytometry we have conclusively demonstrated that anti-angiogenic factors endoglin and Flt-1, implicated in the pathogenesis of PE are associated with STBM, extending the results of Guller et al 2011, showing perfused placenta MV associated Flt-1 and endoglin by single colour flow cytometry, and Rajakumar et al 2012, who demonstrated the release of MV associated sFlt-1 from cultured placental explants by ELISA. Decreased release of endoglin positive STBM from PE placentas and reduced PLAP and endoglin expression on mSTBM, suggests STB dysfunction affects surface antigen expression. Tissue disruption and increased STB enzyme expression [40], [41] associated with mSTBM production and PE placentas could lead to increased release of enzymes, such as MMP-14 that cleaves the extracellular domain of endoglin forming soluble endoglin [41]. Therefore, as well as MV associated endoglin, cleaved placental surface endoglin may also add to the increase in circulating levels associated with PE [42]. The mechanism of release of the GPI anchored PLAP is poorly understood and reports of circulating PLAP levels in PE variable [43], [44]. We however have shown a consistent decrease in PLAP expression on PE placenta derived STBM by flow cytometry and WB suggesting that PLAP is not an appropriate marker for the detection of circulating STBM in PE.

As well as surface Flt-1, perfusion and mSTBM contain several isoforms of sFlt-1, the quantities of which are increased in PE derived STBM. Soluble Flt-1 is therefore released from STB as both intravesicular and membrane bound forms. The sFlt-1 isoforms found in the present study mirror those found by Rajakumar et al 2012 in both normal pregnancy and PE plasma and placental explant ultracentrifuged pellet. Endoglin expression was also higher on PE placenta derived mSTBM. The extent to which placenta derived MV associated proteins reflect protein composition of the source STB was not investigated, but it has been established that placental production and expression of soluble Flt-1 and endoglin is increased by hypoxia and in PE placentas [45]. This would suggest that the increased endoglin and sFlt-1 associated with STBM measured by flow cytometry and WB respectively, is reflective of the placenta from which the STBM originated.

With the demonstration that STBM have the ability to bind the proangiogenic factors VEGF, PlGF and TGFβ and the observation of increased levels of STBM in the circulation of PE women [5]–[7], this constitutes a significant capacity of STBM to bind VEGF, PlGF and TGFβ. Indeed, our observations that addition of VEGF, PlGF and TGF reversed the disruptive effects of mSTBM on cultured HUVEC monolayers demonstrates further the potential sequestering and inhibition of growth factors vital for the maintenance of the vascular endothelium by STBM and also extends previously published results showing that inhibition of endothelial tube formation by MV released from PE placental explant can be reversed by VEGF supplementation [15]. Furthermore, sFlt-1 targeted treatments as a therapy for PE, such as VEGF supplementation or reduction of circulating sFlt-1 levels have shown alleviation of symptoms in animal models of PE [46]–[48]. The question remains as to the biological significance of MV associated and soluble endoglin and Flt-1. The potential for STBM to accumulate in filtering organs such as the liver and spleen [49], [50], implicates MV delivery of antiangiogenic factors in the organ endothelial dysfunction intrinsic to the maternal syndrome of PE.

The growing number of factors associated with STBM suggests that they are complex entities with the potential to affect multiple biological systems. The balance between the immunosuppressive exosomes and proinflammatory MV gives an additional dimension to the role of syncytiotrophoblast derived vesicles during normal pregnancy and PE. As circulating biopsies of the placenta, the characterisation of STBM is essential to better understand, not only the biological effects but also their potential as prognostic and diagnostic biomarkers for early detection of PE. Also, the release of significant quantities of placental MV carrying biologically active moieties broadens the maternal-fetal interface beyond the uterus and into the maternal circulation.
